# Accuracy of calcified plaque volumes by AI-QCT coronary CT angiography: phantom and non-contrast CT verification

**DOI:** 10.1093/ehjimp/qyag018

**Published:** 2026-02-19

**Authors:** Francesca Calicchio, Shawn Newlander, Eric Hu, Elizabeth Epstein, Eric Gros, George Wesbey

**Affiliations:** Division of Radiology and Cardiology, Scripps Health, 9888 Genesee Ave, La Jolla, CA 92037, USA; Division of Radiology and Cardiology, Scripps Health, 9888 Genesee Ave, La Jolla, CA 92037, USA; Division of Radiology and Cardiology, Scripps Health, 9888 Genesee Ave, La Jolla, CA 92037, USA; Division of Radiology and Cardiology, Scripps Health, 9888 Genesee Ave, La Jolla, CA 92037, USA; Medical Physics Department, Iterative Health, 14 Arrow St Floor 3, Cambridge, MA 02138, USA; Medical Physics Department, Iterative Health, 14 Arrow St Floor 3, Cambridge, MA 02138, USA

Accurate quantification of coronary artery calcification (CAC) is essential for risk stratification. The pre-contrast CAC Agatston score (AS) is widely used but lacks SI units. Conventional fixed-threshold volumetric scoring (CVS, mm³, HU ≥130) systematically overestimates total calcified plaque volume (TCPV) because of blooming and beam hardening.^1–5^ Adaptive threshold calcium volume (AVC) applies an FDA-approved algorithm to adjust thresholds and reduce this overestimation.^4^ Reporting TCPV from the entire coronary tree on post-contrast coronary CT angiography (CCTA) is increasingly common in trials, often without validation. It remains unclear whether FDA-cleared artificial intelligence–based quantitative CCTA (AI-QCT) plaque software achieves accuracy comparable to phantom-validated pre-contrast CAC using adaptive methods.

We combined phantom and patient datasets to evaluate AI-QCT for TCPV. The study was approved by the local IRB. A 256-slice single-heartbeat volumetric CT scanner was used for all acquisitions. The phantom and patient CAC study (*n* = 25; ESCR-ESTI 2023, Abstract A-386) employed a multi-diameter calcium insert within an anthropomorphic thorax phantom (QRM),^1,4^ scanned at 120 kVp CAC protocol with and without high definition (HD). Patient CAC was acquired with the same protocol. TCPV was compared using CVS and AVC.

The CCTA study (SCCT 2024, Abstract #50) analysed 20 of the same 25 individuals (40% women, mean age 71, mean CAC 590, heart rate 63) who underwent pre-contrast standard CAC (AVC and CVS) and post-contrast CCTA. AI-QCT TCPV was measured at 140 and 100 kVp, two heartbeats apart with identical phase. Five patients were excluded for poor post-contrast image quality. A previous report detailed compositional plaque comparisons between 140 and 100 kVp^6^; here we compared all TCPV measures against AVC and CVS. Correlation and Bland–Altman analyses assessed AI-QCT TCPV relative to pre-contrast AVC and CVS.

**Phantom results:** Adaptive HU analysis reduced overestimation compared with CVS. Mean normalized accuracy (1.0 = perfect) improved with AVC: CVS-HD 1.59 ± 0.22, CVS 1.75 ± 0.53, AVC-HD 0.93 ± 0.19, and AVC 0.81 ± 0.22 (*P* < 0.01). Error magnitude increased with plaque density, CVS overestimating up to 380%.

**Patient results:** The percent difference between CVS and AVC was significantly associated with both CVS volume (*P* = 0.005) and plaque density (*P* < 0.000). Mean combined dose length product (DLP) for the two CCTA scans was 147.6 mGy-cm. AI-QCT TCPV correlated strongly with pre-contrast 120 kVp AVC (*R* = 0.988 at 140 kVp, *R* = 0.970 at 100 kVp; *P* < 0.001). Mean bias of AI-QCT relative to 120 kVp AVC was −23.1 mm³ at 140 kVp and +20.8 mm³ at 100 kVp, whereas AI-QCT minus CVS bias was much larger (−294.9 and −250.9 mm³) (*Figure 1*).

**Figure 1:**
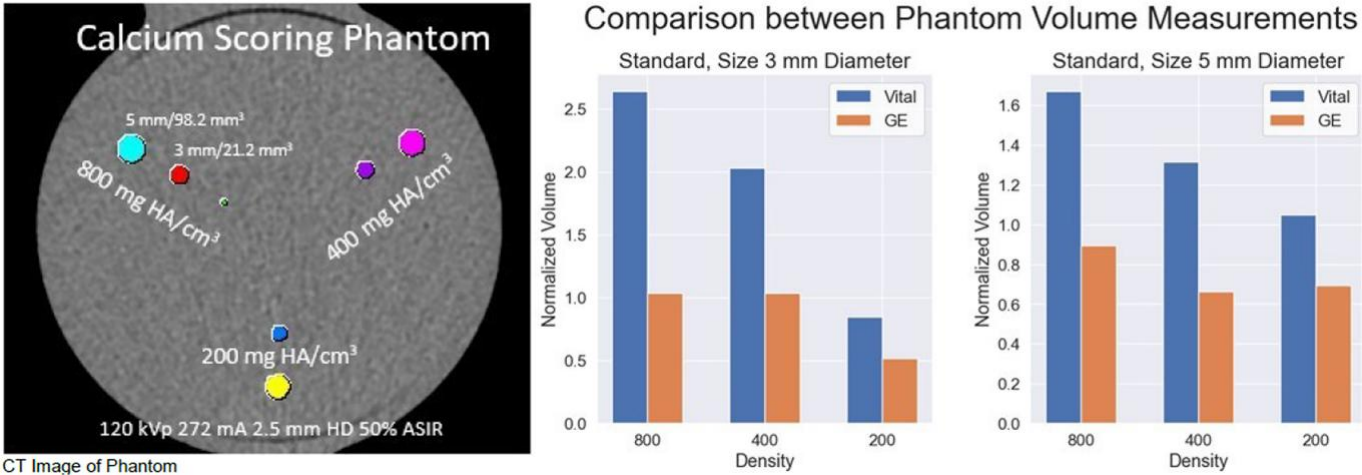
Phantom study (A–D): normalized accuracy and bias for adaptive vs. conventional HU methods across densities.

Calcium attenuates x-rays more strongly at lower energies. Accordingly, 100 kVp studies yield larger TCPV (positive bias) and 140 kVp smaller TCPV (negative bias) compared with 120 kVp AVC. Agreement with adaptive HU volumes remained high at both voltages. In contrast, CVS showed large negative biases at 100 and 140 kVp because of fixed-threshold misclassification: blooming at 100 kVp exaggerates edge voxels but is penalized by the threshold, while reduced blooming at 140 kVp lowers attenuation—yet both produced substantial negative shifts relative to AI-QCT TCPV.

Our prior AI-QCT study at 100 kVp demonstrated excellent scan–rescan precision for TCPV (ICC 0.997; Bland–Altman LOA −4.8, 7.4), non-calcified plaque (0.999; −5.9, 6.8), and total plaque (0.999; −6.4, 9.9).^6^ Here we extend those findings, showing that accurate TCPV by AI-QCT CCTA can be achieved using phantom-validated adaptive methods (*Figure 2*).

**Figure 2:**
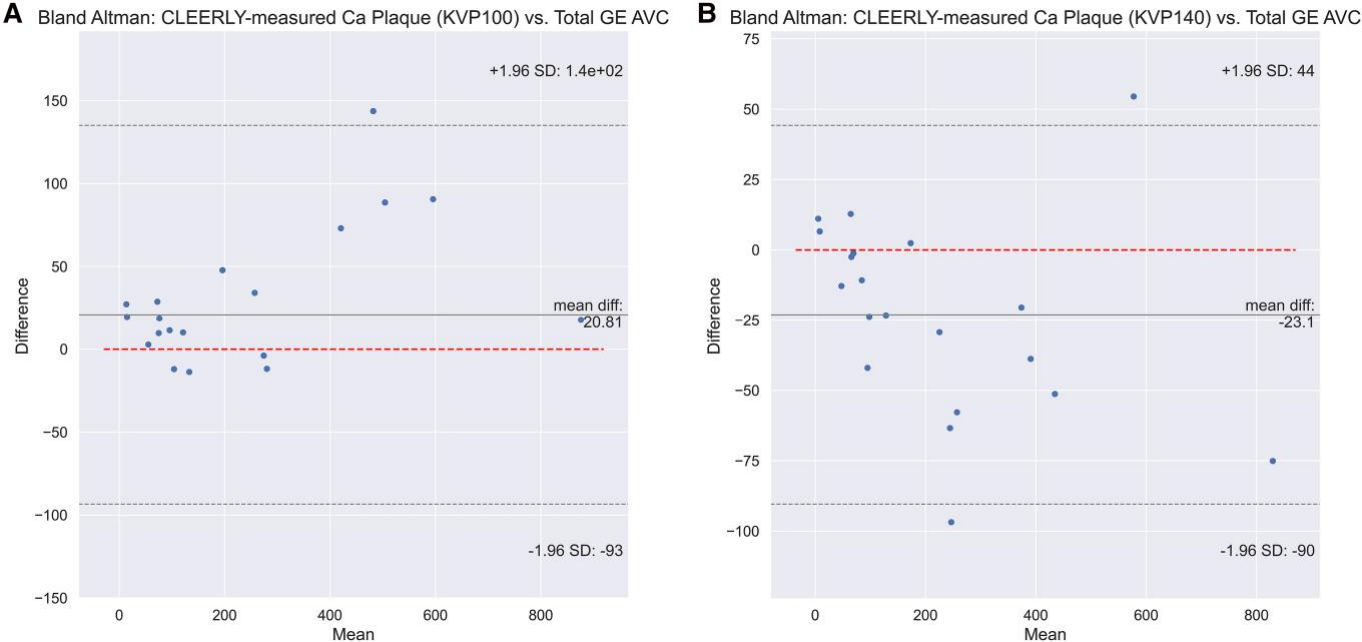
Patient study (E–H): correlation and bland–altman plots comparing AI-QCT TCPV against pre-contrast references. Bland–Altman differences are expressed as AI-QCT TCPV (100 or 140 kVp) (panel A and B)—pre-contrast AVC or CVS (120 kVp).

**Implications:** Despite the retrospective nature and relatively small sample size of this study, our results show that AI-QCT CCTA–derived calcified plaque volumes are accurate when benchmarked to phantom-validated adaptive software. This study does not account for cross-vendor variability, iodine-related spectral effects, or energy-dependent blooming, all of which can influence single energy HU-based plaque measurements in routine clinical practice. While strict standardization of tube voltage and scanner settings remains necessary for current single energy CT workflows, emerging spectral and photon-counting CT approaches that exploit multi-energy information and reference-normalized attenuation may enable more robust plaque quantification under less rigid acquisition conditions in future multi-centre studies.

## Lead author biography


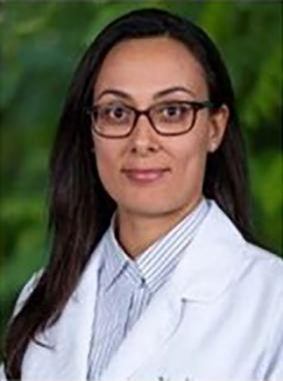


Dr Calicchio stands as a distinguished cardiologist, holding licences in both Italy and the UK. With a robust academic foundation, Dr Calicchio completed her medical education and comprehensive cardiology training at the University of Pisa, Italy, followed by an enriching training at the Royal Brompton Hospital in London, UK. In 2023, Dr Calicchio attained her PhD in ‘Clinical Pathophysiology of Cardiovascular Diseases’ from the University of Pisa. This diverse educational background endows her practice with a breadth of international perspectives and methodologies. Presently, she serves as a cardiology fellow at Southwest Healthcare Medical Education Consortium in California, USA. During her doctoral pursuit, she collaborated closely with the Lundquist Institute at Harbor-UCLA Medical Centre in Torrance, California, USA, where she assumed the role of Research Scholar in 2019, contributing significantly to cutting-edge research initiatives in the field of cardiac CT. Since 2022, Dr Calicchio has held the position of visiting researcher at Scripps Clinic in La Jolla, California, USA, further enriching her research portfolio and expanding her scholarly contributions. Dr Calicchio's research endeavours encompass a wide spectrum, ranging from valvular heart disease to advanced cardiovascular imaging techniques, with a particular emphasis on cardiac CT and echocardiography striving to deepen understanding and pioneer innovative treatments in this domain, thus driving forward the frontiers of cardiovascular medicine.

## Data Availability

Data will be made available upon request subject to institutional approval.
